# Fanconi Anemia: A Rare Genetic Disorder

**DOI:** 10.7759/cureus.38899

**Published:** 2023-05-11

**Authors:** Bharati Thakur, K.M. Hiwale

**Affiliations:** 1 Pathology, Jawaharlal Nehru Medical College, Datta Meghe Institute of Higher Education and Research, Wardha, IND; 2 Pathology, Datta Meghe Institute of Higher Education and Research, Wardha, IND

**Keywords:** autosomal recessive, genetic disorder, pancytopenia, fanconi anemia, café au lait spots

## Abstract

Fanconi anemia is a rare genetic disorder affecting various body systems. Congenital abnormalities, poor hematopoiesis, a higher incidence of acute myeloid leukemia, myelodysplastic syndrome, and malignancies are the hallmarks of this autosomal recessive condition. In certain instances, the clinical signs and highly diverse phenotypic presentation make a diagnosis challenging. In this case report, an eight-year-old boy presented with recurrent episodes of fever, generalized weakness and physical deformities. He had left thumb deformity, triangular face, short stature, and hyperpigmentation with café au lait spots. Bone marrow biopsy revealed hypoplastic marrow, peripheral blood smear revealed pancytopenia, and chromosomal breakage testing was also positive.

## Introduction

Fanconi anemia is an incredibly rare autosomal recessive disease, occurring at a birth rate of one per 350,000 [1]. It was first recognized as a fatal, progressing anemia with brown skin pigmentation in 1927 [1]. In three brothers with abnormalities such as short stature, hypogonadism and skin pigmentation, Fanconi depicted a familial form of aplastic anemia [2]. Fanconi anemia affects one to five people per million people [3]. The Ashkenazi community is more likely to suffer from this illness. Compared to black South Africans, who have a carrier frequency of one in 40,000 [4], Ashkenazi Jews have a disease carrier frequency of one in 89 [5]. Approximately one-third of those who are affected do not have any congenital symptoms of this disease; in this situation, a diagnosis is typically made after the first decade of life, when development abnormalities become obvious [6]. Patients with Fanconi anemia run a greater risk of getting secondary malignancies, like leukemia, squamous cell carcinoma and hepatocellular carcinoma. Both head and neck and anogenital region have a significant risk of developing squamous cell carcinoma [7].

## Case presentation

An eight-year-old male child from India came with recurrent fever episodes, generalized weakness, and physical anomalies presented to the pediatric outpatient department of a tertiary care hospital in central India. All development milestones have been delayed since birth. The patient was the first-born child of consanguineous healthy parents. Antenatally, mother had no specific complaints and was uneventful. Past history of easy bruising and epistaxis was present.

**Figure 1:**
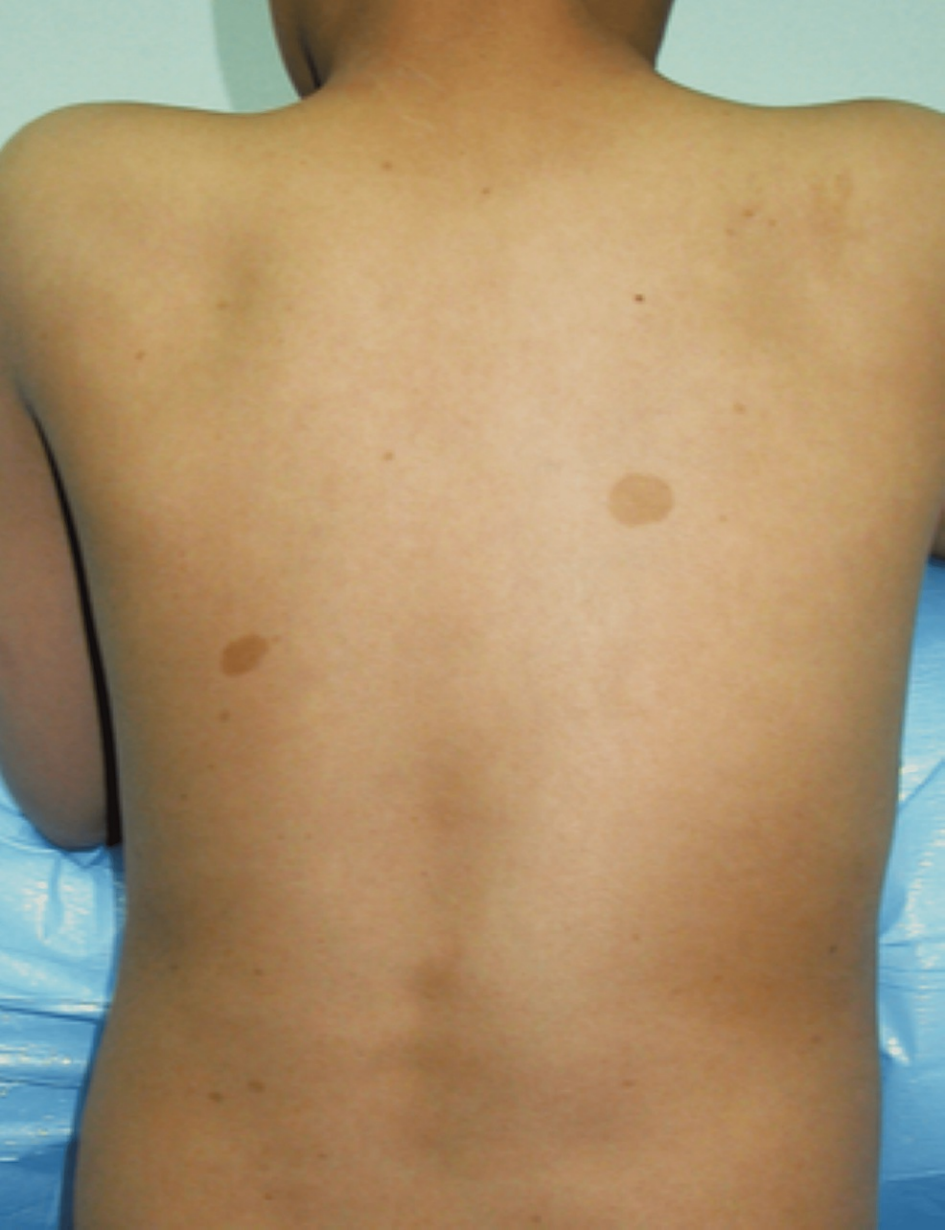
showing café au lait spots in back

**Figure 2:**
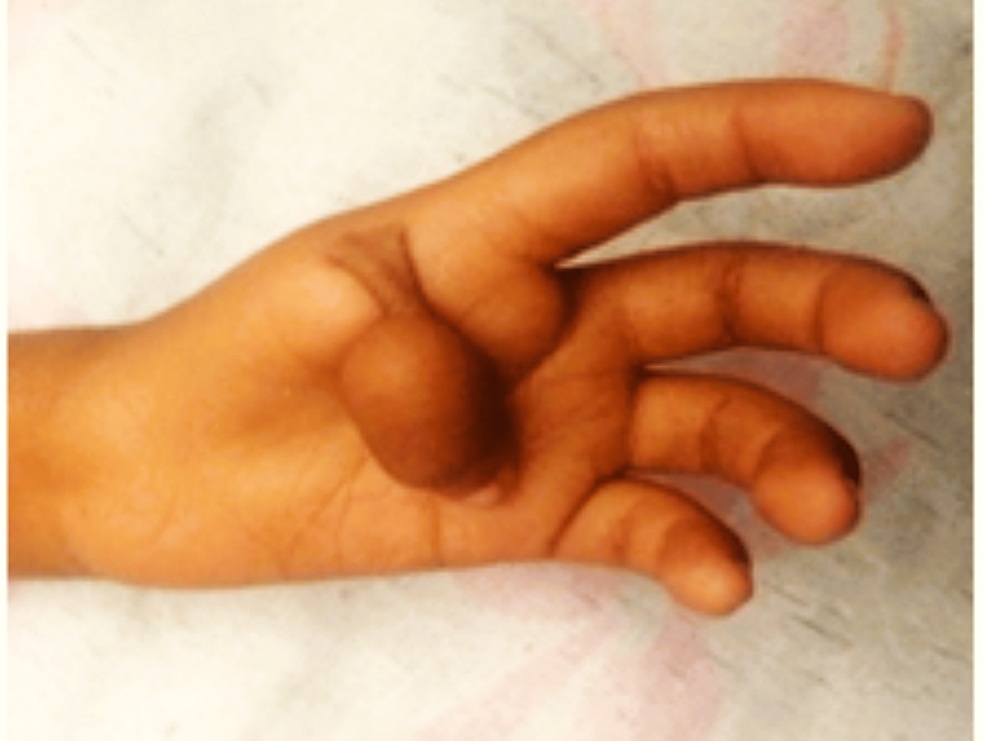
showing left thumb malformation

**Figure 3:**
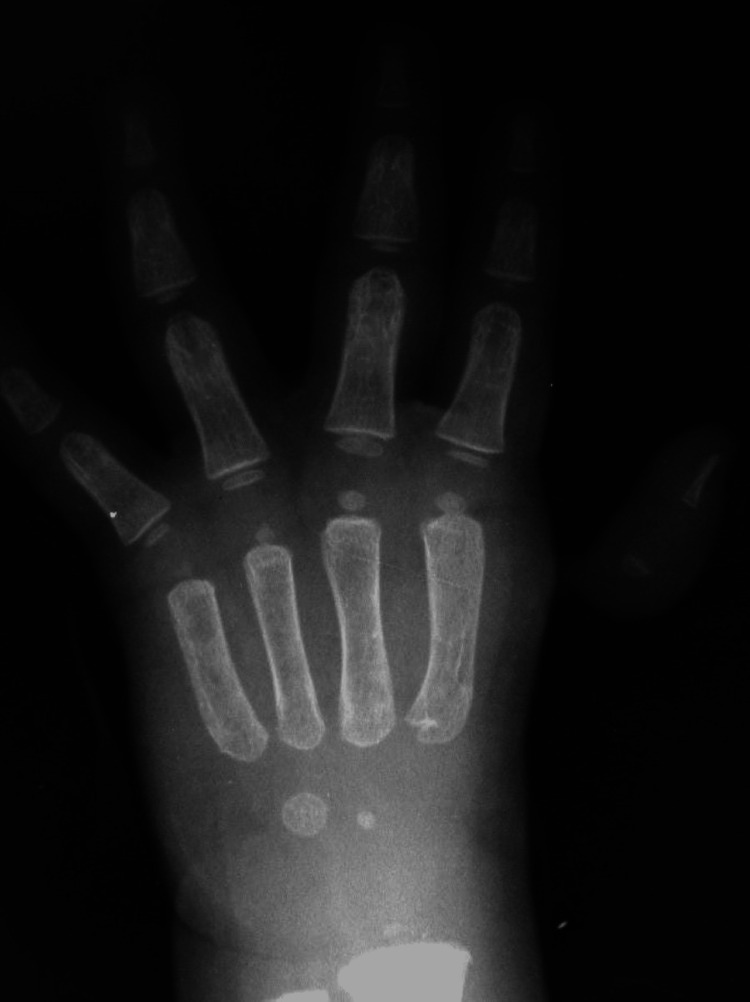
X-ray anterior posterior (AP) view of right hand showing right thumb hypoplasia.

We noted a birth weight of 850 g, spontaneous birth, preterm at 28 weeks based on our observations of the physiological and pathological history. On clinical examination, it was discovered that the patient had a body mass index of the 19th percentile, weight for age percentile is 7%, and was short-statured as height for age was below the 10th percentile as per the Indian Association of Pediatric growth chart, febrile and had a triangular face, café au lait spots on the back (Figure 1) and had a malformed left thumb (Figure 2). Blood tests showed low hemoglobin, total WBC count 2400/cumm, a platelet count of 6000/ml, a normochromic blood picture with thrombocytopenia and pancytopenia, high fetal hemoglobin (HbF) levels in hemoglobin electrophoresis, very high erythropoietin serum level, and mildly raised lactate dehydrogenase (LDH) level. Xray of right hand with anteroposterior view shows right thumb hypoplasia (Figure 3).

**Figure 4:**
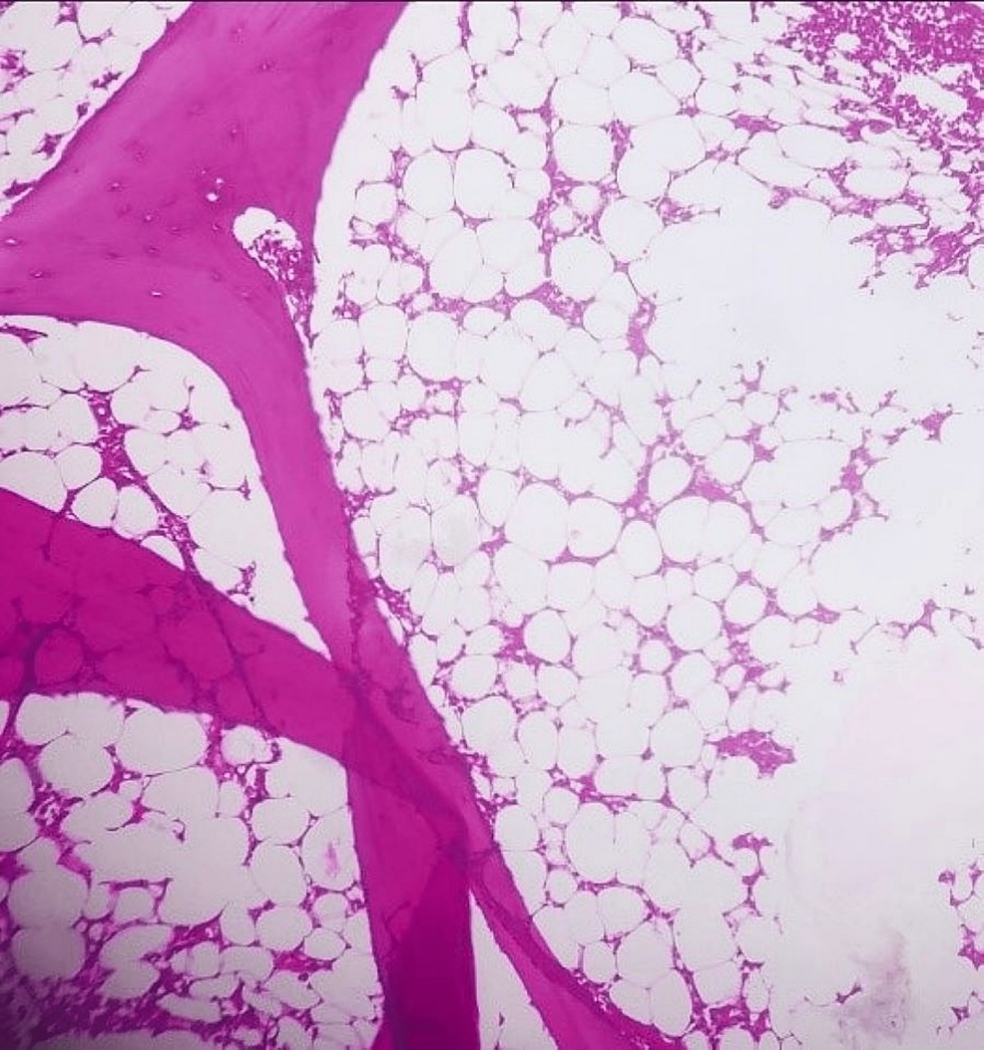
Bone marrow biopsy in Fanconi anemia showing hypocellular marrow, H&E stain

**Figure 5:**
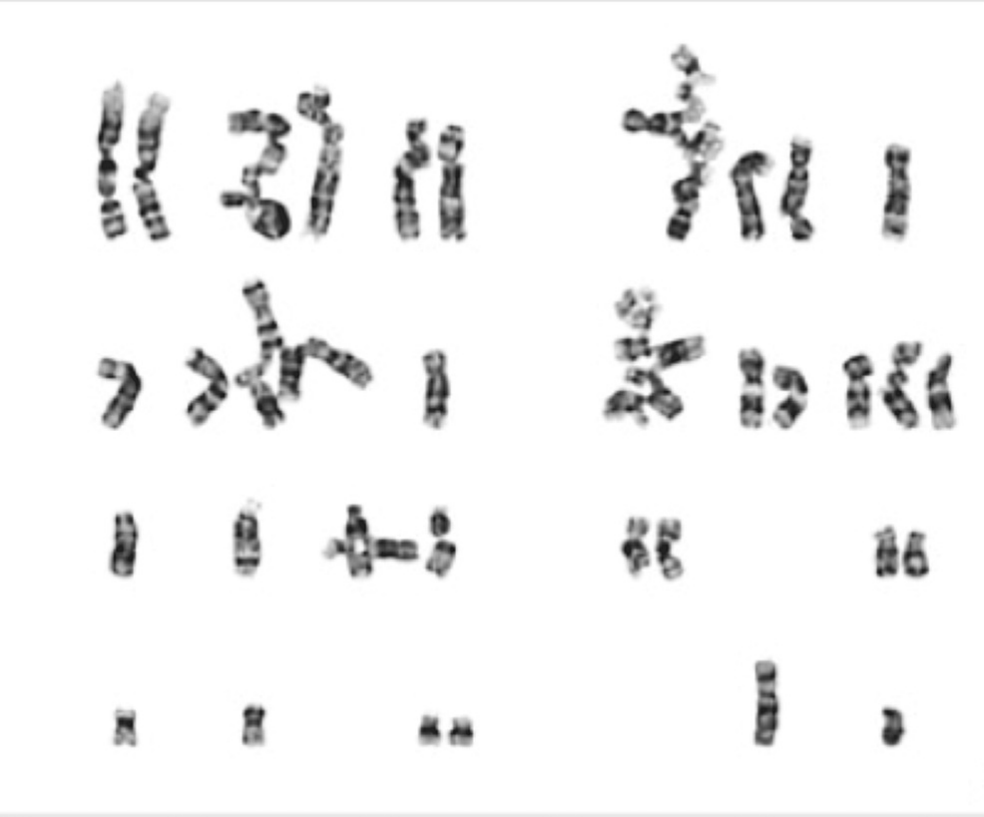
In chromosomal breakage study induced by mitomycin showing part of metaphase spread of Fanconi anemia lymphocyte showing spontaneous chromatid aberration.

To validate the diagnosis, a bone marrow puncture was done, and this revealed a bone with minimal cellularity and diminished hematopoietic components that appeared to be unaltered morphologically (Figure 4). Based on the clinical, laboratory and pathological findings, Fanconi anemia was confirmed. Chromosomal breakage study induces by Mitomycin-C found abnormal for chromosomal breakage syndrome with significant structural aberrations in most of the metaphases studied (Figure 5). Genomic hybridization (aCGH) showed maternally inherited 16q24.3 deletion, including FANCA gene, and next-generation sequencing (NGS) disclosed paternally inherited novel variants in the FANCA gene-Asn1113Tyr and Ser890Asn. The child was referred to another tertiary care for hematopoietic stem cell transplantation.

## Discussion

Guido Fanconi, a Swiss doctor, originally identified an autosomal recessive disease known as Fanconi anemia in 1927 [2]. Fanconi anemia causes prenatal abnormalities, hematological issues, and a propensity for malignancies [8]. Fanconi anemia has been found in a variety of ethnic groups [1]. The frequency of heterozygous carriers is approximately one case per 300 people, while the Fanconi anemia affects one to five per one million people. Occurrence of disease in male and female ratio is equal [2].

Classic phenotype of heterogenous recessive disorder Fanconi anemia is characterized by short stature, malformed thumbs, café au lait spots, microcephaly and a distinctive facial feature such as epicanthal folds, broad nasal base, and micrognathia. Hypogonadism and renal abnormalities are typically present in neonates with Fanconi anemia. Certain cancers, such as acute myeloid leukemia and less frequently liver tumors, mouth cancer, tongue cancer, genital cancer, and brain tumors, are more common in some Fanconi anemia patients [1].

Differential diagnosis for Fanconi anemia can be thrombocytopenia-absent radius (TAR) syndrome, VATER syndrome i.e., affected parts of the body including vertebrae, anus, heart, trachea, esophagus, kidney and limbs (VACTERAL association) and Holt-Oram syndrome. At birth, early signs of TAR syndrome with radial ray defects and severe thrombocytopenia with bleeding manifestations are visible [9]. TAR syndrome patients have been known to exhibit bone deformities, microcephaly and short webbed neck. If the radii are affected in Fanconi anemia, the thumbs are usually absent or hypoplastic; however, in TAR radii are missing but the thumbs are always present. The present case had a malformed thumb that was compatible with the Fanconi anemia diagnosis. Trisomy 18 is rarely associated with radial ray abnormalities or eye anomalies. But trisomy 18 has a wide range of physical anomalies and unusual features [1].

Fanconi anemia manifests as multiple systems-affecting physical abnormalities, and because hematologic abnormalities at birth are so rare, diagnosing Fanconi anemia requires a high threshold of suspicion. Fanconi anemia requires early diagnosis because long-term survival depends on the age at which hematopoietic abnormalities or cancers first manifest. If Fanconi anemia is discovered in the pre-anemic stage, life expectancy can be increased by avoiding drugs and unexpected environmental anomalies associated with malignancy or acquired aplastic anemia [1].

In Fanconi anemia patient frequently needs transfusions of red blood cells. Androgens to increase hemoglobin and platelet count. Fanconi anemia can also be treated with granulocyte colony stimulating factor and granulocyte-macrophage colony stimulating factor for neutropenia. For patients with Fanconi anemia who experience bone marrow failure, the only option for treatment is a bone marrow transplant. If either procedure enables Fanconi anemia patients to live longer, they may now be at an even higher risk for solid tumors, which appear to be the disease of the older patient [10].

Prenatal testing and family planning are two ways to take preventive action. Fetal ultrasound examination, molecular genetic investigation via amniocentesis, and chromosomal breakage tests using diepoxybutane/mitomycin C are all included in prenatal testing [8]. Early diagnosis also provides alternatives for planning a subsequent pregnancy, because of the ability of umbilical cord blood for stem cell transplantation. Currently, bone marrow or umbilical cord blood transfusion from an identical sibling is the recommended method of treating Fanconi anemia [1].

Mutational information is a prerequisite for genetic counselling of family members, screening of potential bone marrow donors who are phenotypically and hematologically normal, and prediction of clinical prognosis on the basis of genotype-phenotype correlations. We describe the successful application of genetic testing to the molecular diagnosis of Fanconi anemia, in a patient and his family.

## Conclusions

A range of congenital abnormalities may coexist with Fanconi anemia, which is a heterogeneous disorder. Aplastic anemia, myelodysplasia, acute myeloid leukemia, and solid malignancies in older ages are the primary causes of morbidity and mortality. It is advised to follow up frequently to check for any emerging cancers. Early discovery will help with the selection of the best course of treatment, the scheduling of genetic counselling, and the selection of a suitable donor for hematopoietic stem cell transplantation.
